# 4Kscore as a Pre-MRI Reflex Test for Men with PSA 1.80–2.99 ng/ml in the Göteborg-2 Prostate Cancer Screening Trial

**DOI:** 10.1016/j.euros.2026.10.001

**Published:** 2026-10-22

**Authors:** Fredrik Möller, Andreas Josefsson, Marianne Månsson, Sigrid Carlsson, Hans Lilja, Vasiliki Spyratou, Jonas Wallström, Jonas Hugosson, Rebecka Arnsrud Godtman

**Affiliations:** a Department of Urology, Institute of Clinical Science, Sahlgrenska Academy, University of Gothenburg, Gothenburg, Sweden; b Department of Urology, Skaraborg Skövde Hospital, Region Västra Götaland, Skövde, Sweden; c Wallenberg Center for Molecular Medicine, Umeå University, Umeå, Sweden; d Department of Urology and Andrology, Institute of Surgery and Perioperative Sciences, Umeå University, Umeå, Sweden; e Division of Clinical Epidemiology of Early Cancer Detection, German Cancer Research Center, Heidelberg, Germany; f Department of Translational Medicine, Division of Urological Cancers, Lund University, Lund, Sweden; g Departments of Pathology and Laboratory Medicine, Surgery (Urology), and Medicine (GU-Oncology), Memorial Sloan Kettering Cancer Center, New York, USA; h Department of Translational Medicine, Lund University, Malmö, Sweden; i Department of Pathology, Sahlgrenska University Hospital, Gothenburg, Sweden; j Department of Radiology, Institute of Clinical Science, Sahlgrenska Academy, University of Gothenburg, Gothenburg, Sweden; k Department of Radiology, Sahlgrenska University Hospital, Region Västra Götaland, Gothenburg, Sweden; l Department of Urology, Sahlgrenska University Hospital, Region Västra Götaland, Gothenburg, Sweden

**Keywords:** Prostate cancer, Diagnosis, Blood markers, Screening

## Abstract

**Background:**

Lowering the prostate-specific antigen (PSA) cut-off from 3.0 to 1.8 ng/ml in prostate cancer (PCa) screening would improve diagnostic yield but substantially increase the number of men undergoing diagnostic work-up.

**Objective:**

To evaluate 4Kscore as a reflex test for pre-magnetic resonance imaging (MRI) risk stratification among men with PSA 1.80–2.99 ng/ml.

**Design, setting, and participants:**

This diagnostic biomarker study was nested within the population-based Göteborg-2 PCa screening trial and included men with PSA ≥1.80 ng/ml who were referred for MRI and provided blood for biomarker research, followed by targeted biopsy if MRI-positive (Prostate Imaging Reporting and Data System [PI-RADS] ≥3). The analysis included 555 men (median age 59 yr) with PSA 1.80–2.99 ng/ml, pre-MRI 4Kscore, and assessable MRI.

**Outcome measurements and statistical analysis:**

The primary outcome was International Society of Urological Pathology (ISUP) grade ≥2. Diagnostic performance of 4Kscore as a pre-MRI reflex test was assessed using sensitivity, specificity, predictive values, and area under the receiver operating characteristic curve (AUC), with 95% confidence intervals (CI).

**Results and limitations:**

MRI was positive in 116/555 men (21%). Overall, PCa was detected in 52/555 men (9.3%), including 27/555 men (4.9%) with ISUP grade ≥2. A 4Kscore ≥7.5% would detect 56% of the ISUP grade ≥2 cancers and reduce detection of ISUP grade 1 by 64%. This approach would reduce MRI referrals by 71% and referrals to biopsy by 67% compared with performing MRI in all men with PSA ≥1.80 ng/ml and performing biopsies in all men with suspected lesions. With a 4Kscore cut-off of 7.5%, sensitivity and specificity for detecting ISUP grade ≥2 were 56% (95% CI = 35–74) and 72% (95% CI = 68–76), respectively. The AUC was 0.69 (95% CI = 0.58–0.79). The limited number of clinically significant PCa cases restricts generalizability.

**Conclusions:**

Using a 4Kscore 7.5% cut-off as a reflex test before MRI in men with PSA 1.80–2.99 ng/ml could substantially reduce referrals to MRI and biopsy, reduce ISUP grade 1 cancer detection, and enable earlier detection of about half of ISUP grade ≥2 cancers. Many men with ISUP grade ≥2 cancers had 4Kscores <7.5%, and most men selected for MRI had PI-RADS <3. Evaluation of lower 4Kscore cut-offs, longer follow-up, and larger prospective studies is required before clinical implementation.

## Take Home Message

Using 4Kscore as a pre-magnetic resonance imaging (MRI) reflex test in men with PSA 1.80–2.99 ng/ml reduces MRI use, biopsies, and low-risk cancer detection, while enabling earlier detection of about half of clinically significant cancers. Larger studies with longer follow-up are needed.

ADVANCING PRACTICE **What does this study add?**

This study provides the first prospective evidence from a population-based screening trial evaluating the 4Kscore as a pre-magnetic resonance imaging (MRI) reflex test in men with prostate-specific antigen (PSA) 1.80–2.99 ng/ml. The findings show that 4Kscore-based risk stratification can identify a subset of men below the conventional PSA cut-off for MRI referral in whom MRI may enable earlier detection of clinically significant prostate cancer. At the prespecified 7.5% cut, approximately 30% of men would be referred for MRI, detecting 55% of International Society of Urological Pathology (ISUP) grade ≥2 cancers at the index screening round while substantially reducing MRI use, biopsies, and detection of low-grade prostate cancer. Many ISUP grade ≥2 cancers had 4Kscores <7.5%, and most men selected for MRI had Prostate Imaging Reporting and Data System <3. Evaluation of lower 4Kscore cut-offs, longer follow-up and larger prospective studies are required before clinical implementation.

**Clinical Relevance**

Optimizing prostate cancer screening requires identifying the most effective and cost-efficient diagnostic algorithm, rather than relying on a single PSA threshold or fixed diagnostic pathway. Incorporating novel biomarkers such as 4Kscore, and other emerging risk-stratification tools may help better select men for MRI and biopsy, thereby reducing unnecessary investigations while maintaining detection of clinically significant disease. Prospective evaluation of different combinations and sequencing of PSA, biomarkers, MRI, and biopsy will be essential to define the optimal screening strategy and maximize both effectiveness and cost-effectiveness. Associate Editor: Roderick C.N. van den Bergh.

**Patient Summary**

Lowering the prostate-specific antigen (PSA) cut-off in prostate cancer screening may detect more cancers, but it also increases the number of magnetic resonance imaging (MRI) scans, biopsies, and detection of low-risk tumors that may never cause harm. In this study, we tested whether 4Kscore, a blood-based test that combines four markers and age, could help select which men with PSA 1.80–2.99 ng/ml should undergo MRI. Using 4Kscore, about three out of 10 men would be referred for MRI. This approach would find about half of the potentially harmful cancers earlier, while avoiding many unnecessary MRI scans and biopsies.

## 1 Introduction

Prostate-specific antigen (PSA) screening followed by biopsies in men with PSA ≥3 ng/ml reduces prostate cancer (PCa) mortality but leads to overdiagnosis [1]. The PCa mortality reduction was only 16% among attenders in the ERSPC study [2], indicating that many men, despite participating in the screening program, are diagnosed too late for cure and eventually die from PCa. Results from the Göteborg-2 trial reported that most men diagnosed with advanced or very high-risk disease at re-screening had a PSA <3.0 ng/ml at the previous screening round, suggesting that a lower PSA cut-off could benefit selected men [3]. The Prostate Cancer Prevention Trial, the Stockholm3 study, the Göteborg-2 trial, and the Barcode1 study have shown that the presence of clinically significant cancers (ISUP grade ≥2) is not infrequent in men with PSA <3.0 ng/ml [4], [5], [6], [7]. However, lowering the PSA cut-off within the large group of men with PSA <3.0 ng/ml would be highly resource-intensive, prompting urgent requests for additional risk stratification [6].

We recently reported that using a 4Kscore cut-off ≥7.5% as a reflex test in men with PSA ≥3.0 ng/ml reduced the need for magnetic resonance imaging (MRI) by 41% and biopsy by 28%, resulting in a 23% reduction of insignificant PCa diagnoses while delaying detection of significant PCa in only 4% of men [8]. However, the diagnostic value of the 4Kscore in men with PSA <3.0 ng/ml is far less established, particularly in a screening setting.

Rather than lowering the cut-off for biopsy for all men with PSA <3 ng/ml, an alternative strategy would be to use reflex testing of additional biomarkers within this PSA range to enable earlier detection of clinically significant PCa without substantially increasing overdiagnosis or resource use. We hypothesize that a pre-MRI 4Kscore can identify a subset of men with PSA 1.80–2.99 ng/ml in whom MRI would allow earlier detection of clinically significant disease, while detection of most clinically insignificant cancers would be deferred. The PSA interval of 1.80–2.99 ng/ml corresponds to the lower cut-off used in one randomized arm of the Göteborg-2 trial.

The primary aim of this study was to evaluate whether use of a pre-MRI 4Kscore as a reflex test in men with PSA between 1.80–2.99 ng/ml can identify a subgroup of men in whom MRI may enable earlier detection of clinically significant PCa, while limiting unnecessary investigations and overdiagnosis.

## 2 Patients and methods

### 2.1 Design

The Göteborg-2 biomarker study (G2B) is nested within the Göteborg-2 trial, a population-based randomized screening study. In short, the Göteborg-2 trial randomly selected approximately 38 000 men aged 50–60 yr from the Swedish Population Register and invited them to participate in PSA based screening; by June 1, 2024, 55% of the invited men had attended at least one screening round. Men who opted to participate were randomized into one of the three study arms with different PSA thresholds, followed by MRI and MRI-targeted biopsies if MRI was positive (Prostate Imaging Reporting and Data System (PI-RADS) ≥ 3). Details about the Göteborg-2 trial study design are described in previous papers [9], [10].

Participants with PSA exceeding the cut-off received a written invitation to participate in G2B. Following informed consent, participants were asked to provide a separate blood sample for the 4Kscore analysis at the time of the MRI. Participation was limited because of restricted availability of study nurses and logistical challenges with biobanking during the peak of the coronavirus disease 2019 pandemic. Therefore, not all eligible males were invited to participate. The G2B study was approved by the regional ethical board (988-15 and T294-18) as an auxiliary study to the Göteborg-2 trial.

This analysis included men in the study arm with a PSA cut-off ≥1.80 ng/ml for MRI referral. Eligible men had PSA levels between 1.80 and 2.99 ng/ml before June 1, 2024 (allowing inclusion from screening rounds one to five, as men in the Göteborg-2 study were reinvited to repeated screening rounds depending on prior screening results), had an assessable MRI, and had provided written informed consent for participation in the G2B study. Men with a previous biopsy in the Göteborg-2 trial and those who had a positive MRI but refused biopsy were excluded.

4Kscore was calculated from a blood sample provided at the time of MRI according to a prespecified algorithm [11], [12] based on age and analysis of the serum concentrations of total PSA, free PSA, intact PSA, and human kallikrein-related peptidase 2, yielding a risk of diagnosis of clinically significant PCa [13].

### 2.2 Statistical analysis

The primary objective of this study was to evaluate the performance of 4Kscore as a pre-MRI reflex test in men with PSA in the range 1.80–2.99 ng/ml. Clinically significant PCa was defined as International Society of Urological Pathology (ISUP) grade ≥2. For 4Kscore cut-offs ranging from 0% to 20%, sensitivity, specificity, negative predictive value (NPV), and positive predictive value (PPV) were calculated with 95% confidence intervals (CIs) for detecting clinically significant PCa. Men with a 4Kscore below the cut-off, or above the cut-off but with negative MRI findings, were classified as test-negative. Men with a 4Kscore above the cut-off and positive MRI findings were classified as test-positive. Since only MRI-positive men underwent biopsy, estimates of sensitivity, specificity, NPV, and PPV should be interpreted in the context of the MRI-based diagnostic pathway rather than as biopsy-verified outcomes for the entire screening cohort. The overall diagnostic performance of 4Kscore as a reflex test was assessed using the area under the receiver operating characteristic curve (AUC) with 95% CI. In addition, decision-curve analysis was performed to evaluate the net benefit of 4Kscore as a reflex test.

To illustrate the clinical consequences of using 4Kscore as a reflex test before MRI among men with PSA 1.80–2.99 ng/ml, results were presented as the number of saved MRIs, biopsies, and number of prostate cancers detected per 1000 men, using the prespecified cut-off of 7.5% and exploratory cut-offs of 5%, 6%, 9%, 10%, and 15%. Because the 4Kscore was measured in a separate blood sample from the screening PSA, a sensitivity analysis excluded men with PSA <1.8 ng/ml or ≥3.0 ng/ml in the blood sample used for the 4Kscore testing to account for within-person PSA variability. In addition, a sensitivity analysis evaluated PSA density for biopsy decision-making in PI-RADS 3 lesions.

Statistical analysis was performed in SPSS version 29 and R Statistical Software, version 4.5.1.

## 3 Results

### 3.1 Study population

Between October 2015 and June 2024, 6954 men participated in the Göteborg-2 trial arm with a PSA cut-off ≥1.80 ng/ml, of whom 1494 had PSA levels between 1.80 and 2.99 ng/ml at least once. Of these men, 571 men were included in the G2B study; however, 16 were excluded due to non-assessable MRI, earlier biopsy in the Göteborg-2 study, or refusal to undergo biopsy. A total of 555 men were included in the final analysis (Fig. 1).

**Fig. 1:**
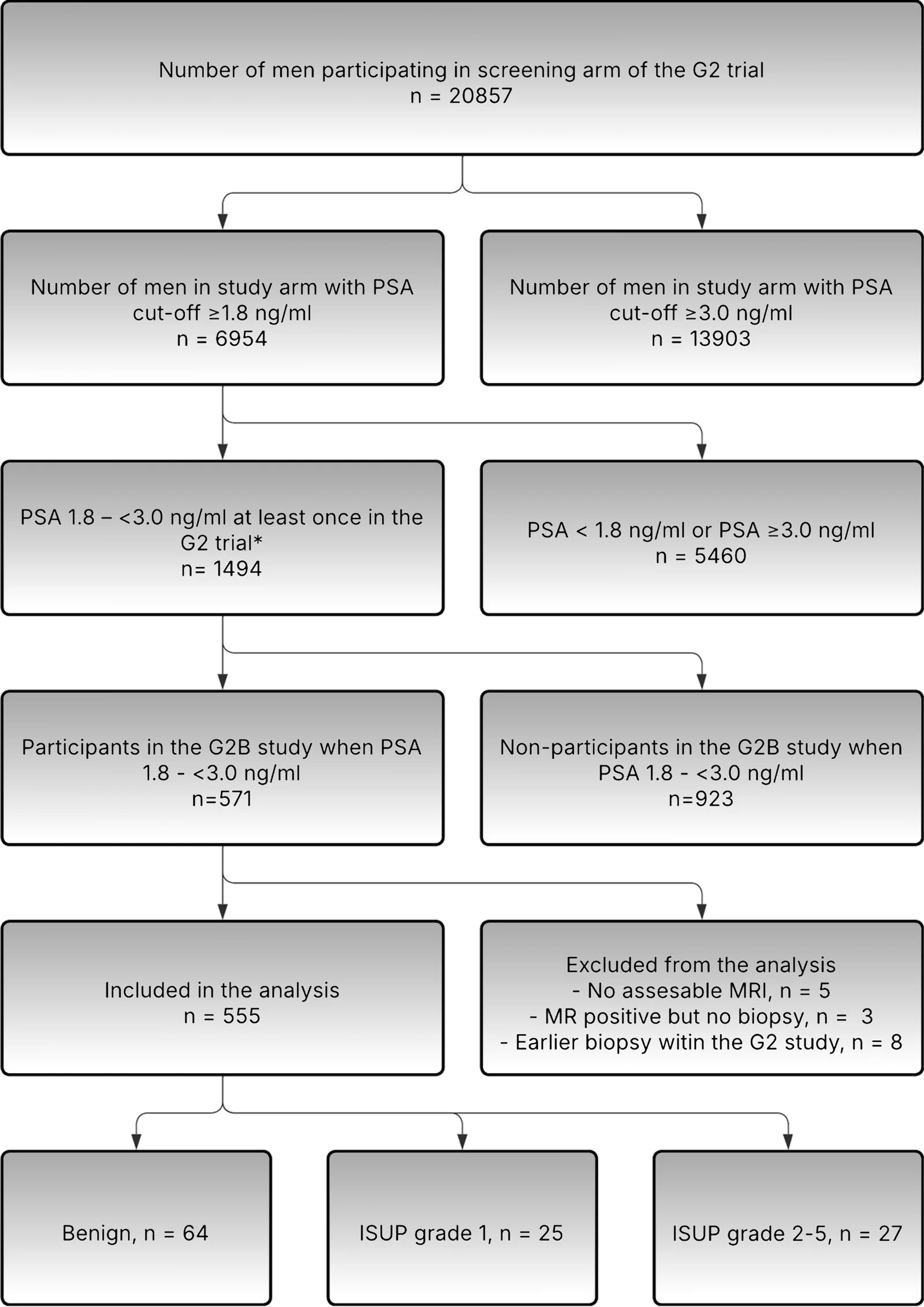
Study flowchart. G2 = Göteborg-2 biomarker; PSA = prostate-specific antigen; MRI = magnetic resonance imaging; ISUP = International Society of Urological Pathology. * Participants may originate from different screening rounds.

Baseline characteristics for participants and non-participants in the G2B study were largely similar, as presented in Table 1. Overall, 116/555 (21%) G2B participants had a positive MRI. Among these men, 64/116 (55%) had benign biopsies, 25/116 (22%) had ISUP grade 1 cancer, and 27/116 (23%) had ISUP grade ≥2.

**Table 1:** Baseline characteristics of Göteborg-2 biomarker study participants and non-participants with PSA 1.80–2.99 ng/ml, and outcomes of MRI and targeted biopsies

	**Participants in the study**	**Non-participants in the study**
**Number of participants, n**	555	835
**Age (yr), median (IQR)**	59 (56–62)	59 (55–62)
**PSA (ng/ml), median (IQR)**	2.1 (1.9–2.4)	2.1 (1.9–2.5)
**Prostate volume (ml), median (IQR)**	40 (33–48)	41 (34–51)
**PSA density (ng/ml^2^), median (IQR)**	0.05 (0.04–0.07)	0.05 (0.04–0.06)
**PI-RADS, n (%)**		
1–2	439 (79)	674 (81)
3	50 (9.0)	70 (8.4)
4	60 (11)	84 (10)
5	6 (1.1)	7 (0.8)
**MRI result, n (%)**		
Negative	439 (79)	674 (81)
Positive	116 (21)	161 (19)
**Biopsy result, n (% of positive MRI)**		
Benign	64 (55)	75 (47)
ISUP grade 1	25 (22)	59 (37)
ISUP grade 2	22 (19)	23 (14)
ISUP grade ≥3	5 (4.3)	4 (2.5)

PSA = prostate-specific antigen, PSAD = prostate-specific antigen density, PI-RADS = Prostate Imaging-Reporting and Data System, MRI = magnetic resonance imaging, ISUP = International Society of Urological Pathology; IQR = interquartile range.

### 3.2 4Kscore distribution

The median 4Kscore in the study population was 5.2% (interquartile range [IQR] 3.6–8.0). The distribution of 4Kscores among men with MRI-positive examinations was 47/116 (41%) for 4Kscores <5%, 30/116 (26%) for 4Kscores between 5% to <7.5%, 21/116 (18%) for 4Kscores between 7.5% to <10%, and 18/116 (16%) for 4Kscores ≥10%. The proportion of ISUP grade ≥2 among MRI-positive men increased with higher 4Kscore, from 6/47 (13%) among men with 4Kscores <5%, 6/30 (20%) for 4Kscores 5% to <7.5%, 6/21 (29%) for 4Kscores 7.5% to <10%, to 9/18 (50%) among those with 4Kscores ≥10% (Table 2).

**Table 2:** 4Kscore in men with PSA 1.80–2.99 ng/ml: MRI outcomes by PI-RADS score and targeted biopsy results by ISUP grade

	**4Kscore**				
	**0.0 to <5.0%**	**5.0 to <7.5%**	**7.5 to <10.0%**	≥**10.0**%	**Total**
PI-RADS, n (%)	*n* = 259	*n* = 135	*n* = 81	*n* = 80	*n* = 555
1–2	212 (82)	105 (78)	60 (74)	62 (78)	439 (79)
3	20 (7.7)	15 (11)	9 (11)	6 (7.5)	50 (9.0)
4	26 (10)	14 (10)	10 (12)	10 (13)	60 (11)
5	1 (0.4)	1 (0.7)	2 (2.5)	2 (2.5)	6 (1.1)
ISUP grade, n (% of MRI- positive)	*n* = 47	*n* = 30	*n* = 21	*n* = 18	*n* = 116
Benign	33 (70)	16 (53)	12 (57)	3 (17)	64 (55)
1	8 (17)	8 (27)	3 (14)	6 (33)	25 (22)
2	5 (11)	5 (17)	4 (19)	8 (44)	22 (19)
≥3	1 (2.1)	1 (3.3)	2 (9.5)	1 (5.5)	5 (4.3)

PI-RADS = Prostate Imaging-Reporting and Data System, MRI = magnetic resonance imaging, ISUP = International Society of Urological Pathology.

Among men with negative MRI (PI-RADS ≤2), the median 4Kscore was 5.1% (IQR = 3.6–7.8). In MRI-positive men, the median 4Kscore was 4.7% (3.0–7.4) for benign biopsies, 5.8% (4.7–9.8) for ISUP grade 1, 8.6% (5.0–12.3) for ISUP grade 2, and 7.6% (6.6–9.9) for ISUP grade ≥3**.** Overall, ISUP grade ≥2 was diagnosed in 27/116 men (22 ISUP grade 2, two ISUP grade 3, and three ISUP grade 4 cases). Of these, 12/27 (44%) had a 4Kscore <7.5%. ISUP grade ≥3 cancer occurred in 2/77 (2.6%) men with a 4Kscore <7.5% (Supplementary Table 1)

#### 3.2.1 4Kscore performance

The diagnostic performance of 4Kscore thresholds from 0–20% for the diagnosis of ISUP grade ≥2 is presented in Fig. 2. At the prespecified cut-off of 7.5%, the PPV was 9% (95% CI = 6–13), NPV 97% (95% CI = 95–98), sensitivity 56% (95% CI = 35–74), and specificity 72% (95% CI = 68–76). Analyses of additional cut-offs are presented in Supplementary Tables 2 and 4.

**Fig. 2:**
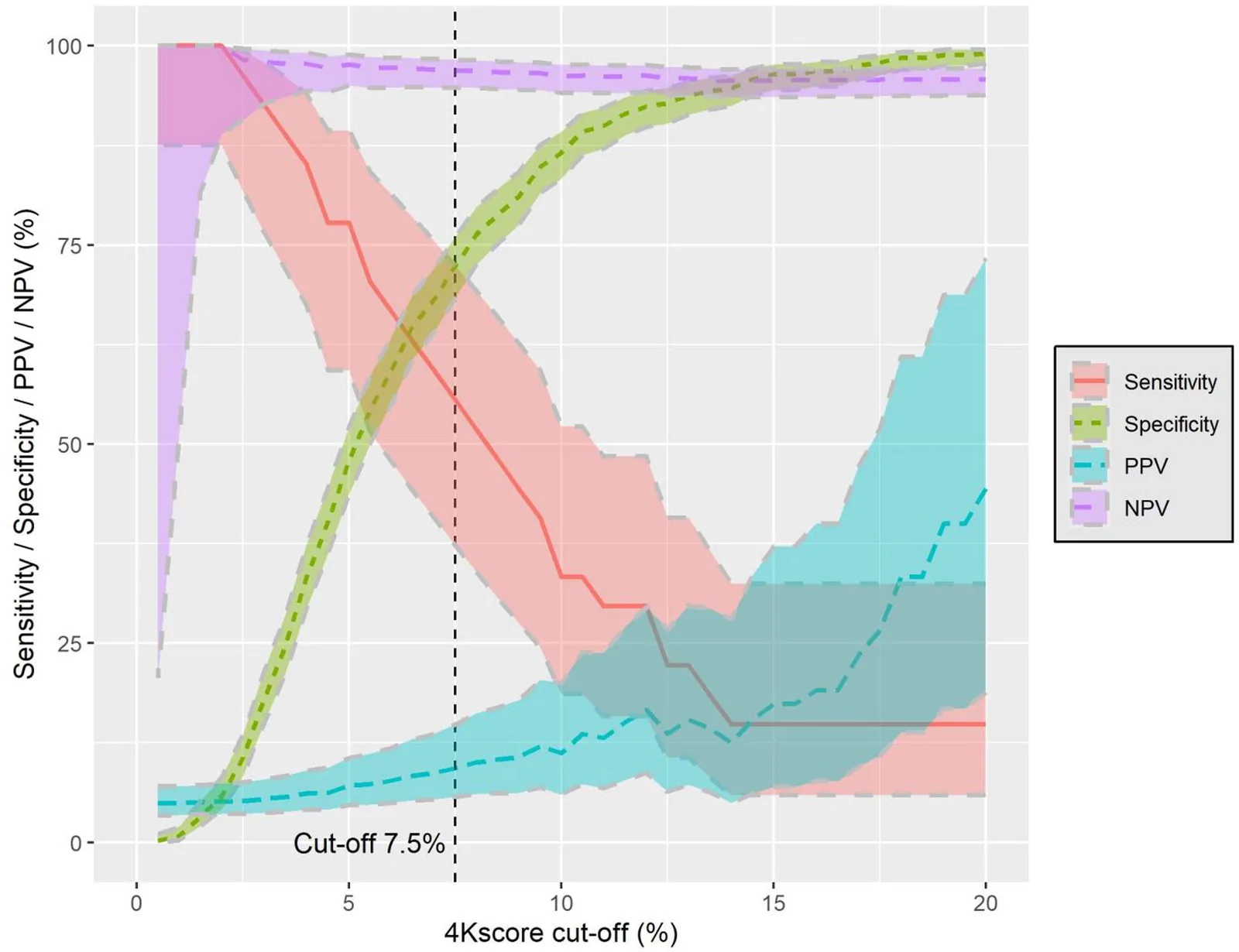
Sensitivity, specificity, PPV, and NPV at different 4Kscore cutoffs for the diagnosis of clinically significant prostate cancer (ISUP grade ≥2). The shaded area represents the 95% confidence interval. The dashed vertical line represents 7.5% 4Kscore cutoff. PPV = Positive predictive value, NPV = Negative predictive value, ISUP = International Society of Urological Pathology.

The AUC was 0.69 (95% CI = 0.58–0.79) for the 4Kscore as a reflex test before MRI for the detection of ISUP grade ≥2. The decision-curve analysis demonstrated that including 4Kscore as a reflex test had a greater net benefit compared with performing MRI on all men or MRI on no men, for threshold probabilities of 4–9% (ISUP grade ≥2). The net benefit reached a maximum improvement of 0.14 compared with performing MRI on either all or none, with this maximum attained at threshold of 5% (Supplementary Fig. 1).

### 3.3 Clinical implications

The clinical consequences of applying 4Kscore cut-offs of 5% and 7.5%, respectively, as a reflex test prior to MRI are presented in Table 3. A 4Kscore cut-off of 7.5% would result in approximately 29% of men undergoing MRI, to find 56% of the ISUP grade ≥2 cancers at an earlier time point compared to waiting for the standard cut-off of 3.0 ng/ml at the index screening round. In a sensitivity analysis excluding 220 men with PSA <1.8 ng/ml or PSA ≥3.0 ng/ml at the time of MRI from the serum sample used for the 4Kscore testing, all ISUP grade ≥3 cancers were detected (Supplementary Table 3). In a sensitivity analysis incorporating PSA density (≥0.1 ng/ml^2^) for biopsy decisions in PI-RADS 3 lesions, 4Kscore cut-offs of 7.5% reduced biopsies by 78% and detected approximately the same number of ISUP grade ≥2 cancers (Supplementary Table 5).

**Table 3:** Detection of prostate cancer and reduction in MRI scans/biopsies per 1000 men with PSA 1.8–2.99 ng/ml, using 4Kscore cut-offs of 5% and 7.5% as reflex tests, followed by MRI and targeted biopsy for PI-RADS ≥3

**Per 1000 men**	**PSA 1.80–2.99 ng/ml for diagnostic work-up**	**PSA 1.80–2.99 ng/ml and 4Kscore ≥5% for diagnostic work-up**		**PSA 1.80–2.99 ng/ml and 4Kscore ≥7.5% for diagnostic work-up**	
		Performed	Avoided/delayed (%)	Performed	Avoided/delayed (%)
MRI	1000	533	467 (47)	290	710 (71)
Negative MRI	791	409	382 (48)	220	571 (72)
Positive MRI	209	124	85 (41)	70	139 (67)
Targeted biopsies	209	124	85 (41)	70	139 (67)
Benign	115	56	59 (51)	27	88 (77)
ISUP grade 1	45	31	14 (31)	16	29 (64)
ISUP grade ≥2	49	38	11 (22)	27	22 (44)
ISUP grade ≥3	9	7	2 (22)	5	4 (44)

PSA = prostate-specific antigen, MRI = magnetic resonance imaging, ISUP = International Society of Urological Pathology.

Values in parentheses in the avoided/delayed columns represent the percentage reduction relative to the reference strategy of MRI in all men with PSA 1.80–2.99 ng/ml followed by targeted biopsies for PI-RADS ≥3.

## 4 Discussion

In this screening-based diagnostic biomarker study, we evaluated the utility of 4Kscore as a reflex test before MRI among men with PSA 1.80–2.99 ng/ml. Using the prespecified 4Kscore cut-off 7.5%, would markedly reduce MRI use by 71%, biopsies by 67%, and detection of ISUP grade 1 cancers by 64%. The 4Kscore as a pre-MRI reflex test still detected three of five ISUP grade ≥3 cancers and about half of ISUP grade ≥2 cancers at the index screening round. This strategy could enable risk stratification within a group conventionally considered below the cut-off for diagnostic work-up by identifying men with PSA <3.0 ng/ml and elevated 4Kscore who may benefit from earlier detection, as opposed to lowering the PSA cut-off for all men, which would substantially increase resource use and overdiagnosis. Thus, rather than aiming to detect all clinically significant cancers in a single screening round by lowering the cut-off to 1.8 and performing MRI on all these men, this approach enables prioritization of earlier detection for a subset of higher-risk individuals, while deferring lower-risk cases to later rounds in a repeated screening setting.

Many clinically significant cancers not detected at the index screening round using a 4Kscore cut-off of 7.5% were Gleason score 3 + 4 with limited pattern 4. However, there were some clinically significant cancers that demonstrated adverse pathological features, including two ISUP grade ≥3 cancers and two cases with pT3 disease at radical prostatectomy. However, the optimal timing of PCa diagnosis remains uncertain. Whether earlier detection at lower PSA levels translates into improved curability remains uncertain and will be evaluated in planned follow-up analyses within the Göteborg-2 trial.

This analysis was nested within a screening trial including 555 men with PSA 1.80–2.99 ng/ml, all of whom were referred to MRI and provided informed consent to contribute blood for 4Kscore analysis. Of these men, 21% underwent targeted biopsies based on suspicious findings (PI-RADS 3–5) at MRI. Our evaluation should be interpreted within the constraints of that context. The 4Kscore showed moderate discrimination (AUC 0.69, 95% CI = 0.58–0.79) for distinguishing men with and without clinically significant PCa detected by MRI-targeted biopsies. Decision-curve analysis indicated that using 4Kscore as a pre-MRI reflex test provided a small net clinical benefit over performing MRI on all men or MRI in no men within a limited range of thresholds (4–9%) for clinically significant PCa. The largest improvement was attained at threshold 5%. These findings suggest that a 4Kscore pre-MRI reflex test may be most useful in a cautious diagnostic strategy, where MRI should be performed even when suspicion of clinically significant cancer is low. This possibly points to a lower 4Kscore cut-off than the 7.5% cut-off used in this study. MRI positivity varied little across different 4Kscore values, whereas the prevalence of clinically significant PCa increased with higher 4Kscore values, underscoring the 4Kscore’s ability to provide a meaningful risk gradient even if its additional value is limited within an MRI-guided pathway.

Interestingly, our prior Göteborg-2 analysis of 4Kscore as a reflex test in men with PSA ≥3 ng/ml demonstrated better overall performance [8]. Several possible factors may explain the weaker performance observed in the narrow range of PSA 1.8–3.0 ng/ml. First, the lower AUC likely reflects restricted risk variation and reduced biological separation between men with and without clinically significant PCa, resulting in lower discrimination at fixed cut-offs. PCa at lower PSA levels generates more subtle kallikrein elevations, either because the tumors are smaller or because they release lower amounts of kallikreins relative to tumor volume. Future analyses will explore whether circulating kallikrein levels are associated with tumor volume or Gleason grade. Second, because the 4Kscore model has been calibrated largely in populations with higher PSA, it may underestimate absolute risk in this lower PSA range. Together, these observations could indicate a need for recalibrating the 4Kscore for PSA <3.0 ng/ml or adopting a lower cut-off in this population.

Underdiagnosis in men with low PSA remains a concern. Most men with PSA <3.0 ng/ml have negative findings on MRI, yet some harbor high-risk prostate cancer. The rationale for lowering the PSA threshold in screening is to identify these high–risk cancers earlier and improve the chance of cure. Importantly, several studies suggest that high-grade PCa presenting at low PSA levels may be associated with increased prostate cancer-specific mortality [14], [15]. In the first screening round of the Göteborg-2 trial, 4.5% of MRI-positive men with PSA 1.8–3.0 ng/ml had ISUP grade ≥3 cancer [6]. However, lowering the PSA cut-off from 3.0 to 1.8 ng/ml substantially increased MRI demand 2.6-fold and overdiagnosis of low–risk tumors 2.3-fold. In our study, a lower 4Kscore cut-off of 5% would detect four of five ISUP grade ≥3 cancers while still halving MRI use and reducing overdiagnosis of low-risk disease by 31%, suggesting that a lower 4Kscore may offer a more balanced use of MRI resources in men with low PSA.

4Kscore performance has been evaluated in screening settings [12], [16]. In the ProtecT cohort, Bryant et al [12] reported that among men with PSA ≥3 ng/ml, a 4Kscore ≥6% reduced detection of low-risk tumors by 35%, while delaying diagnosis of clinically significant cancer in 11% of cases. Using the same cut-off, we observed a larger proportion of clinically significant PCa not detected (35%), consistent with the lower discrimination and lower prevalence in the PSA 1.8–3.0 ng/ml range. The 4Kscore has also been evaluated for long-term prognostic outcomes. Among men with PSA ≥2.0 ng/ml, the 15-yr PCa-specific mortality risk was 1.7% for 4Kscore <7.5% compared with 13% for 4Kscore ≥7.5% [17]. Collectively, these data suggest that men with low PSA levels and a 4Kscore <7.5% constitute a low-risk group with a favorable PCa prognosis.

Other biomarkers may also be considered. Stockholm3, a blood test, which combines protein, genetic, and clinical variables, has been evaluated in a screening setting. Studies indicate that using Stockholm3 before MRI in men with PSA ≥1.5 ng/ml increases detection of clinically significant cancer but also increases detection of low-risk tumors [5]. However, these comparisons were made against a higher PSA (3.0 ng/ml), and the added value of Stockholm3 at the same PSA level remains unclear. Long-term follow-up from the STHLM3 trial further suggests that men with PSA 1–3 ng/ml and elevated Stockholm3 scores are at increased risk of clinically significant PCa and biochemical recurrence, supporting the potential value of improved risk stratification within this PSA range [18]. Alternative risk-stratified screening approaches independent of PSA are under evaluation. In the BARCODE1 trial, polygenic risk score–based screening identified clinically significant PCa in men below conventional PSA-based referral thresholds but was also associated with a substantially higher rate of detecting ISUP grade 1 cancer compared with the Göteborg-2 and ProScreen trials [7], [19].

The 4Kscore is currently being evaluated prospectively in the ProScreen trial using a stepwise screening approach, combining PSA, 4Kscore, and MRI among men with PSA ≥3 ng/ml [20]. Extending screening to lower PSA levels would substantially increase MRI demand, while access to MRI and experienced radiologists remains limited in many health care systems. In this context, 4Kscore may serve as a capacity-preserving approach at low PSA, balancing resource use against the proportion of clinically significant PCa detected versus delayed to subsequent screening rounds.

The main strength of this study is the evaluation of the 4Kscore within a population-based cohort in which all included men underwent MRI independently of 4Kscore result, with systematic and standardized interpretation of MRI scans and biopsies. The primary limitation is the small number of clinically significant prostate cancer cases (*n* = 27), restricting the statistical precision and generalizability of the results. To illustrate the uncertainty of the estimates, approximately 2500 participants would be required to achieve a 95% CI width of approximately 0.1 for the AUC, underscoring the need for larger prospective studies. The proportion of PCa was somewhat higher among non-participants, which may also have influenced diagnostic performance estimates. In addition, although comparison with PSA density is clinically relevant, PSA density in the present study was based on MRI-derived prostate volume and therefore cannot serve as a true pre-MRI reflex test. PSA density was therefore not included in the prespecified analysis plan. However, in a post hoc sensitivity analysis, PSA density was evaluated as a post-MRI biopsy-decision tool for PI-RADS 3 lesions. Nevertheless, because no formal comparator analysis was performed, the added value of 4Kscore over PSA density and other simpler risk-stratification approaches remains uncertain. The cost-effectiveness of 4Kscore-based reflex testing should also be evaluated before implementation in screening. Finally, 4Kscore was measured at the MRI visit and was therefore based on a separate blood sample from the screening PSA. This could have introduced misclassification around the PSA threshold given within-person PSA variability, particularly at low PSA levels [21]. However, excluding men with PSA <1.8 ng/ml or ≥3.0 ng/ml at the MRI-visit sample did not substantially change the overall results. Importantly, in this sensitivity analysis, no ISUP grade ≥3 cancers were delayed at either 5% or 7.5% 4Kscore cut-offs, suggesting that PSA variability around the PSA cut-off may have contributed to some of the apparent 4Kscore failures observed in the primary analysis.

## 5 Conclusions

In prostate cancer screening, the use of the 4Kscore as a pre-MRI reflex test in men with PSA levels of 1.80–2.99 ng/ml substantially reduces MRI use, biopsies, and detection of low-grade cancers, suggesting that 4Kscore-based risk stratification may improve the feasibility of lower PSA-threshold screening. This strategy enables selective earlier detection of about half of the clinically significant cancers at the index screening round. Larger studies with longer follow-up are needed before clinical implementation to determine whether men with clinically significant PCa and low 4Kscore values have a lower risk of progression to metastatic or lethal disease.

***Author contributions:***

Fredrik Möller had full access to all the data in the study and takes responsibility for the integrity of the data and the accuracy of the data analysis.

*Study Conception and design:* Möller, Josefsson, Hugosson, Hellström, Godtman, Månsson.

*Acquisition of data:* Möller, Josefsson, Månsson, Spyratou, Wallström, Hellström, Lilja, Godtman, Hugosson.

*Analysis and interpretation of data*: Möller, Josefsson, Hugosson, Godtman, Månsson.

*Drafting of the manuscript:* Möller.

*Critical revision of the manuscript for important intellectual content:* All Authors.

*Statistical analysis:* Möller, Månsson.

*Obtaining funding:* Josefsson, Hugosson, Lilja, Carlsson, Möller.

*Administrative, technical, or material support:* None.

*Supervision:* Arnsrud Godtman.

*Other*: None.

  ***Financial disclosures:*** Fredrik Möller certifies that all conflicts of interest, including specific financial interests and relationships and affiliations relevant to the subject matter or materials discussed in the manuscript, are the following: Rebecka Arnsrud Godtman has received lecture fees from Ipsen and Bayer on several occasions. Hans Lilja is named on a patent for a statistical method to detect prostate cancer commercialized as the 4Kscore test by OPKO Health with rights to receive royalties from sales of the test, and has stock in OPKO Health. Hans Lilja is named on a patent on free PSA antibodies as theranostics for prostate cancer licensed to Diaprost AB with rights to receive licensing income from this relationship, serves on the Scientific Advisory Board for Fujirebio Diagnostics Inc., owns stock in Arctic Partners, Diaprost AB, and Acousort AB.

  ***Funding/Support and role of the sponsor:*** The Göteborg 2 study was funded by Karin and Christer Johansson’s Foundation, the Swedish Cancer Society, the County Council for West Sweden, the Swedish Research Council, the Swedish Prostate Cancer Foundation, and Kunt and Alice Wallenberg Foundation. Fredrik Möller’s work was supported by the Research Foundation at Skaraborg Hospital. The work of Hans Lilja was supported in part by a Cancer Center Support Grant to MSKCC (P30-CA008748) and U01-CA266535 from the National Institutes of Health / National Cancer Institute, and support from the Swedish Cancer Society 23 3075 Pj 01 H and Prostate Cancer Foundation. The funding providers had no access to the study database and had no role in data collection, management, analysis, manuscript preparation, or the decision to publish.

  ***Acknowledgments:*** We thank all participants in the Göteborg-2 prostate cancer screening trial. We sincerely thank the study nurses Jenny Fernström, Ann Carlstrand, Ümmügülsüm Kibar Yakici, and Anna Richardsson for recruiting participants and supporting all protocol activities, Jonas Laurila Bergman for assistance in establishing the biorepository for the G2B study, and Hélen Ahlgren and Maria Nyberg for their administrative support and data management for the study. This study was conducted using professional biobank services from Biobank West and Biobank Sweden. We are further grateful to Mona Hassan Al-Battan, in Dr. Lilja’s laboratory at Lund University, for performing the kallikrein marker measurements.

  ***Declaration of Generative AI and AI-assisted technologies in the writing process:*** During the preparation of this work, the author(s) used OpenAI/ChatGPT in order to improve the language and readability. After using this tool/service, the author(s) reviewed and edited the content as needed and take(s) full responsibility for the content of the published article.
