# Supplementary material for: 4Kscore as a Pre-MRI Reflex Test for Men with PSA 1.80–2.99 ng/ml in the Göteborg-2 Prostate Cancer Screening Trial
Source: Eur Urol Open Sci. 2026 Oct 22;94:43–51. doi: 10.1016/j.euros.2026.10.001 (PMC13718394; doi:10.1016/j.euros.2026.10.001)
Supplement: Supplementary file 1 [file mmc1.docx]

**Supplementary Material for “4Kscore as a Pre-MRI Reflex Test for Men with PSA 1.80–2.99 ng/ml in the Göteborg-2 Prostate Cancer Screening Trial”**

**Supplementary Table 1.** Clinical characteristics of men with clinically significant prostate cancer and PSA 1.8-3 ng/ml and 4Kscore < 7.5%

**Supplementary Table 2.** Sensitivity, specificity, PPV, and NPV at different 4Kscore cutoffs for the diagnosis of clinically significant prostate cancer (ISUP grade ≥2) at different 4Kscore cut-offs.

**Supplementary Table 3.** Sensitivity analysis excluding 220 men with PSA <1.8 ng/ml or PSA ≥3 ng/ml at the time of 4K testing (based on the blood sample collected at the MRI visit) was performed.

**Supplementary Table 4.** Detection rates of prostate cancer and avoided MRI scans/biopsies at different 4Kscore cut-offs as pre-MRI reflex test, per 1,000 men with PSA 1.8–3.0 ng/ml.

**Supplementary Table 5.** Sensitivity analysis incorporating PSA density for biopsy decisions in PI-RADS 3 lesions.

**Supplementary Figure 1.** Decision curve analysis.

**Supplementary table 1**

Clinical characteristics of men with clinically significant prostate cancer and PSA 1.8-3 ng/ml and 4Kscore < 7.5%

| **4Kscore (%)** | **PSA at screening (ng/ml)** | **PSA at MRI (ng/ml)** | **T-stadium** | **PI-RADS** | **Gleason** | **Primary treatment** |
| --- | --- | --- | --- | --- | --- | --- |
| 7.0 | 2.7 | 2.1 | T2 | 5 | 3+4 (grade 4 5-20%) | RP (pT2) |
| 6.6 | 2.5 | 1.8 | T1 | 4 | 4+4 | RP (pT2) |
| 6.1 | 2.0 | 2.8 | T1 | 3 | 3+4 (grade 4 20-50%) | RP (pT3) |
| 5.5 | 1.8 | 2.2 | T1 | 3 | 3+4 (grade 4 5-20%) | RP (pT2) |
| 5.1 | 1.9 | 2.6 | T2 | 4 | 3+4 (grade 4 <5%) | RP (pT2) |
| 5.0 | 2.9 | 4.1 | T1 | 3 | 3+4 (grade 4 5-20%) | AM |
| 4.2 | 2.0 | 1.2 | T1 | 4 | 4+3 | RT |
| 4.1 | 1.9 | 2.0 | T2 | 4 | 3+4 (grade 4 5-20%) | RP (pT2) |
| 3.9 | 2.2 | 2.0 | T1 | 3 | 3+4 (grade 4 <5%) | RT |
| 3.2 | 2.1 | 3.0 | T1 | 5 | 3+4 (grade 4 <5%) | RP (pT3) |
| 2.7 | 1.9 | 2.9 | T1 | 3 | 3+4 (grade 4 <5%) | RP (pT2) |
| 2.4 | 2.0 | 2.6 | T1 | 4 | 3+4 (grade 4 <5%) | AM |

PSA = prostate-specific antigen, MRI = Magnetic Resonance Imaging, PI-RADS = Prostate Imaging-Reporting and Data System, RP=radical prostatectomy, RT=radiotherapy, AM=active monitoring

**Supplementary table 2**

Sensitivity, specificity, PPV, and NPV at different 4Kscore cutoffs for the diagnosis of clinically significant prostate cancer (ISUP grade ≥2) at different 4Kscore cut-offs.

Values are shown as estimates with 95% confidence intervals in parentheses.

| **4Kscore cut-off** | **Sensitivity**  **(95% CI)** | **Specificity**  **(95% CI)** | **PPV**  **(95% CI)** | **NPV**  **(95% CI)** |
| --- | --- | --- | --- | --- |
| **MRI in all men** | 1.00 (0.87 – 1.00) | 0.83 (0.80 – 0.86) | 0.23 (0.16 – 0.32) | 1.00 (0.99 – 1.00) |
| **5%** | 0.78 (0.58 - 0.91) | 0.48(0.44 - 0.52) | 0.07 (0.05 - 0.11) | 0.98 (0.95 - 0.99) |
| **6%** | 0.67 (0.48 - 0.81) | 0.59 (0.55 - 0.63) | 0.08 (0.05 - 0.12) | 0.97 (0.95 - 0.99) |
| **7.5%** | 0.56 (0.35 - 0.74) | 0.72 (0.68 - 0.76) | 0.09 (0.06 - 0.13) | 0.97 (0.95 - 0.98) |
| **9%** | 0.44 (0.28 - 0.63) | 0.81 (0.77 - 0.84) | 0.11 (0.06 - 0.18) | 0.97 (0.94 - 0.98) |
| **10%** | 0.33 (0.19 - 0.52) | 0.87 (0.83 - 0.89) | 0.11 (0.06 - 0.20) | 0.96 (0.94 - 0.98) |
| **15%** | 0.15 (0.06 -0.32) | 0.96 (0.94 - 0.98) | 0.17 (0.07 - 0.37) | 0.96 (0.94 - 0.97) |
| **20%** | 0.15 (0.06 -0.32) | 0.99 (0.98 - 1.00) | 0.44 (0.19 - 0.73) | 0.96 (0.94 - 0.97) |

PPV = Positive predictive value, NPV = Negative predictive value

**Supplementary table 3**

Sensitivity analysis excluding 220 men with PSA <1.8 ng/ml or PSA ≥3 ng/ml at the time of 4K testing (based on the blood sample collected at the MRI visit) was performed.

Detection rates of cancer and avoided MRI scans/biopsies per 1,000 men with PSA 1.80–2.99 ng/mL, using 4Kscore cut-offs of 5% and 7.5% as reflex tests, followed by MRI and targeted biopsy for PI-RADS ≥3.

Values in parentheses in the avoided/delayed columns represent the percentage reduction relative to reference strategy of MRI in all men with PSA 1.80-2.99 ng/ml followed by targeted biopsies for PI-RADS ≥3.

| *Per 1000 men* | *PSA 1.80 – 2.99 ng/ml for diagnostic work-up* | *PSA 1.80 – 2.99 ng/ml and 4Kscore* ≥*5% for diagnostic work-up* | | *PSA 1.80 – 2.99 ng/ml and 4Kscore* ≥*7.5% for diagnostic work-up* | |
| --- | --- | --- | --- | --- | --- |
|  |  | Performed | Avoided/delayed (%) | Performed | Avoided/delayed (%) |
| *MRI* | 1000 | 490 | 510 (51) | 248 | 752(75) |
| *Negative MRI* | 788 | 376 | 412 (52) | 191 | 597 (76) |
| *Positive MRI* | 212 | 114 | 98 (46) | 57 | 155 (73) |
| *Targeted biopsies* | 212 | 114 | 98 (46) | 57 | 155 (73) |
| *Benign* | 125 | 51 | 74 (59) | 18 | 107 (86) |
| *ISUP grade 1* | 39 | 27 | 12 (31) | 15 | 24 (62) |
| *ISUP grade* ≥*2* | 48 | 36 | 12 (25) | 24 | 24 (50) |
| *ISUP grade* ≥*3* | 3 | 3 | 0 (0) | 3 | 0 (0) |

PSA = prostate-specific antigen, MRI = Magnetic Resonance Imaging, ISUP = International Society of Urological Pathology

**Supplementary table 4**

Detection rates of cancer and avoided MRI scans/biopsies per 1,000 men with PSA 1.8–3.0 ng/mL, at different 4Kscore cut-offs as reflex tests, followed by MRI and targeted biopsy for PI-RADS ≥3

Values in parentheses in the avoided/delayed columns represent the percentage reduction relative to reference strategy of MRI in all men with PSA 1.80-2.99 ng/ml followed by targeted biopsies for PI-RADS ≥3.

| per 1000 MRI | MRI | | Biopsies | | Benign biopsies | | ISUP grade 1 | | ISUP grade 2 | | ISUP grade **≥** 3 | |
| --- | --- | --- | --- | --- | --- | --- | --- | --- | --- | --- | --- | --- |
|  | Performed | Avoided (%) | Performed | Avoided (%) | Performed | Avoided (%) | Diagnosed | Avoided (%) | Diagnosed | Delayed (%) | Diagnosed | Delayed (%) |
| MRI all men | 1000 |  | 209 |  | 115 |  | 45 |  | 40 |  | 9 |  |
| 4Kscore cut-off |  |  |  |  |  |  |  |  |  |  |  |  |
| 5% | 533 | 467(47) | 124 | 85 (41) | 56 | 59 (51) | 31 | 14 (31) | 31 | 9 (23) | 7 | 2 (22) |
| 6% | 420 | 580 (58) | 99 | 110 (53) | 45 | 70 (61) | 22 | 23 (51) | 25 | 15 (38) | 7 | 2 (22) |
| 7.5% | 290 | 710 (71) | 70 | 139 (67) | 27 | 88 (77) | 16 | 29 (64) | 22 | 18 (45) | 5 | 4 (44) |
| 9% | 202 | 798 (80) | 56 | 153 (73) | 18 | 97 (84) | 16 | 29 (64) | 18 | 22 (55) | 4 | 5 (56) |
| 10% | 144 | 856 (86) | 34 | 175 (84) | 7 | 108 (94) | 11 | 34 (76) | 14 | 26 (65) | 2 | 7 (78) |
| 15% | 41 | 959 (96) | 9 | 200 (96) | 0 |  | 2 | 43 (96) | 5 | 35 (88) | 2 | 7 (78) |
| 20% | 16 | 984 (98) | 7 | 202 (97) | 0 |  | 0 |  | 5 | 35 (88) | 2 | 7 (78) |

MRI = Magnetic Resonance Imaging, ISUP = International Society of Urological Pathology

**Supplementary table 5.**

Sensitivity analysis incorporating PSA density for biopsy decisions in PI-RADS 3 lesions.

Men with PI-RADS 4–5 lesions proceeded to biopsy irrespective of PSA density, whereas men with PI-RADS 3 lesions proceeded to biopsy only if PSA density was ≥0.10 ng/ml/ml. Detection rates of cancer and avoided MRI scans/biopsies per 1,000 men with PSA 1.80–2.99 ng/mL.

Values in parentheses in the avoided/delayed columns represent the percentage reduction relative to reference strategy of MRI in all men with PSA 1.80-2.99 ng/ml followed by targeted biopsies for PI-RADS ≥3.

| *Per 1000 men* | *MRI in all men, biopsy PI-RADS ≥3* | *MRI in all men, biopsy PI-RADS 4–5 or PI-RADS 3 + PSAD ≥0.10* ng/ml² | | 4Kscore ≥7.5%, biopsy PI-RADS ≥ 3 | | *4Kscore ≥7.5%, biopsy PI-RADS 4–5 or PI-RADS 3 + PSAD ≥0.10* ng/ml² | |
| --- | --- | --- | --- | --- | --- | --- | --- |
|  |  | Performed | Avoided/delayed (%) | Performed | Avoided/delayed (%) | Performed | Avoided/delayed (%) |
| *MRI* | 1000 | 1000 | 0 | 290 | 710 (71) | 290 | 710 (71) |
| *Negative MRI* | 791 | 791 | 0 | 220 | 571 (72) | 220 | 571 (72) |
| *Positive MRI* | 209 | 209 | 0 | 70 | 139 (67) | 70 | 139 (67) |
| *Targeted biopsies* | 209 | 126 | 83 (40) | 70 | 139 (67) | 47 | 162 (78) |
| *Benign* | 115 | 58 | 57 (50) | 27 | 88 (77) | 13 | 102 (89) |
| *ISUP grade 1* | 45 | 29 | 16 (36) | 16 | 29 (64) | 9 | 36 (80) |
| *ISUP grade* ≥*2* | 49 | 40 | 9 (18) | 27 | 22 (44) | 25 | 24 (49) |
| *ISUP grade* ≥*3* | 9 | 9 | 0 | 5 | 4 (44) | 5 | 4 (44) |

MRI = magnetic resonance imaging; PI-RADS = Prostate Imaging–Reporting and Data System; PSAD = prostate-specific antigen density; ISUP = International Society of Urological Pathology.

**Supplementary figure 1**

Decision curve analysis.

Net benefit is defined as the percentage of men with positive MRI and clinically significant prostate cancer detected after taking harm of missing clinically significant prostate cancer into account, and threshold probability is risk of clinically significant prostate cancer for which patient and/or doctor would choose to perform MRI. Net intervention (MRI examinations) avoided is defined as percentage avoided MRI in men with negative MRI or benign biopsy, after taking harm of missing clinically significant prostate cancer into account.

MRI = Magnetic Resonance Imaging,
